# Neutrophil extracellular traps in cancer: not only catching microbes

**DOI:** 10.1186/s13046-021-02036-z

**Published:** 2021-07-14

**Authors:** Livia Ronchetti, Nouha Setti Boubaker, Maddalena Barba, Patrizia Vici, Aymone Gurtner, Giulia Piaggio

**Affiliations:** 1 SAFU Unit, IRCCS - Regina Elena National Cancer Institute, Rome, Italy; 2 Laboratory of proteins engineering and bioactive molecules (LIP-MB), National Institute of Applied Sciences and Technology of Tunis (INSAT), The University of Carthage, Tunis, Tunisia; 3 Division of Medical Oncology 2, IRCCS Regina Elena National Cancer Institute, Rome, Italy; 4 Institute of Translational Pharmacology (IFT), National Research Council (CNR), Rome, Italy

**Keywords:** citH3, PAD4, Chemokine receptors, Neutrophils, PD-L1 inhibitors, NET, Cancer liquid biopsies

## Abstract

Neutrophils are the most abundant type of white blood cells circulating throughout the bloodstream and are often considered the frontline defenders in innate immunity. However, neutrophils are increasingly being recognized as having an important role in tumorigenesis and carcinogenesis due to their aberrant activation by molecules released into the tumor microenvironment. One defensive response of neutrophils that is aberrantly triggered during the neoplastic process is called NETosis, where activated neutrophils expel their DNA and intracellular contents in a web-like structure known as a neutrophil extracellular trap (NET). In cancer, NETosis has been linked to increased disease progression, metastasis, and complications such as venous thromboembolism. NET structures released by neutrophils can also serve as a scaffold for clot formation, shining new light on the role of neutrophils and NETosis in coagulation-mediated diseases.

Here, we review current available knowledge regarding NET and the related NETosis process in cancer patients, with an emphasis on pre-clinical and clinical data fostering the identification and validation of biomarkers of NET with a predictive/prognostic role in cancer patients treated with immunotherapy agents. NETosis biomarkers, e.g., citH3, may integrate correlates of immunogenicity currently available (e.g., PD-L1 expression, TMB, TILs) and help select the subsets of patients who may most benefit from the use of the therapeutic weapons under discussion.

## Background

Neutrophils are the most abundant type of granulocytes comprising of 40 to 70% of all white blood cells in humans and form an essential part in our organism’s initial defense against external pathogens. As a vital part of the innate immune system neutrophils are produced in the bone marrow and are then released into the bloodstream ready to be rapidly recalled to the sites of infection attracted by a chemotactic gradient of chemokines [1]. Besides their role in infections, neutrophils have been shown to be involved in tumor growth and progression, where their recruitment to the microenvironment of different cancers is associated with adverse patient outcomes [2].

**Fig. 1:**
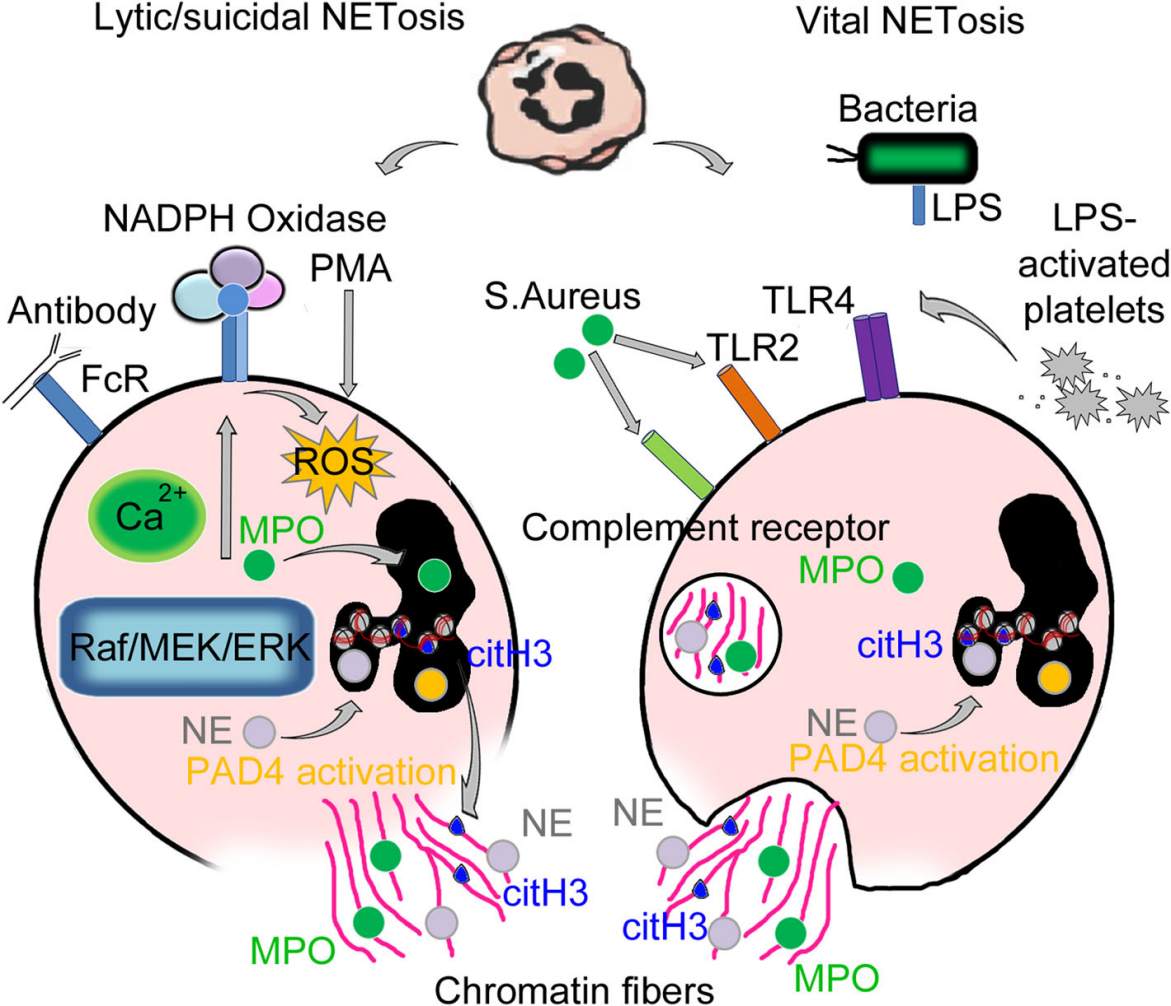
Activating stimuli and molecules involved in the two types of NETosis: lytic NETosis (left) consists in a mechanism that effectively kills neutrophils, which by breaking down releases the filamentous lattice made of decondensed chromatin, histones, and lytic enzymes into the extracellular space. Known activators of lytic/suicidal NETosis are PMA (Phorbol 12-myristate 13-acetate) and antibodies through binding to the Fc receptor; extracellular signals lead to calcium-dependent activation of NADPH oxidase and release of reactive oxygen species (ROS). ROS cause the activation of the PAD4 enzyme and translocation from granules to the nucleus of Neutrophil Elastase (NE) and Myeloperoxidase (MPO); combined action of PAD4, NE, and MPO results in citrullination of histones, in particular H3, and subsequent chromatin decondensation. The nuclear membrane of neutrophils breaks, and the chromatin mixed with enzymes and histones is released first into the cytoplasm and then into the extracellular space, following the rupture of the cell membrane, forming Neutrophil Extracellular Traps (NETs). In vital NETosis (right), the neutrophil remains intact releasing the reticulum via a system of vesicles; the latter mechanism appears to be independent of NADPH oxidase activation. Microbial infections, especially from *S. aureus*, recognized by Toll-Like Receptor-2 (TLR2) or Complement Receptor and LPS-activated platelets that bind Toll-Like Receptor-4 are among the major proven activators of this second pathway

Neutrophils have evolved different strategies to exert their anti-microbial activity, including phagocytosis and degranulation as well as the more recently studied process, called NETosis, which consists in releasing extracellular web-like structures, termed NETs (Neutrophils Extracellular Traps) [3, 4]. NETs consist of decondensed chromatin filaments coated in histones and antimicrobial proteins. Two types of NEtosis have been characterized: “lytic/suicidal” NETosis, a slow cell death pathway and an alternative pathway called “vital” NEtosis which involves a rapid release of NETs into the extracellular space from live cells [5]. The molecular mechanisms and receptors involved in the activation of NETosis are still under investigation, however, some of the molecular pathways that are involved in the two types of NET release have been identified. Lytic/suicidal NETosis is a mechanism that strictly depends on the production of reactive oxygen species (ROS). A number of stimuli, such as phorbol 12-myristate 13-acetate (PMA), antibodies, cholesterol crystals, bacterial lipopolysaccharide LPS, interleukins, have been shown to induce lytic NETosis [6]. The major activators of lytic NETosis in vitro are PMA, a *plant*-derived natural organic compound, and IL-8, even though the role of the latter is more controversial [7]. Another mechanism of activation of lytic NETosis appears to be the binding of antibodies to specific receptors for the Fc region on the plasma membrane of neutrophils [8]. In lytic NETosis Raf/MEK/ERK signal transduction cascade is activated leading to calcium release from the endoplasmic reticulum and then on to subsequent phosphorylation of NADPH oxidase subunits, one of the major sources of ROS [9, 10]. The serine protease neutrophil elastase (NE) and myeloperoxidase (MPO) from cytoplasmatic granules translocate to the nucleus [11] leading to nuclear chromatin decondensation and activation of the peptidyl arginine deiminase 4 (PAD4), a calcium-dependent enzyme that citrullinates histones, in particular H3 (citH3). The nucleus loses the classical lobular shape and the nuclear membrane breaks down; the decondensed chromatin is therefore released in the cytosol [12]; the plasma membrane disintegrates so that decondensed chromatin associated with granular proteins and histones are released as reticular structures outside the dying cell [13] (Fig. 1).

Vital NETosis is induced by bacterial infections, particularly those due to *S. aureus*, through the interaction with Toll-like Receptor 2 (TLR2) and Complement Receptors [6, 7, 14]. The LPS of the outer membrane of Gram negative bacteria, is able to induce NETosis directly, or indirectly through activated platelets. Indeed, the recognition of LPS by the Toll-like Receptor 4 (TLR4) on the surface of the platelets, activates the latter, which in turn act on neutrophils, consequently inducing NETosis [15]. Vital NETosis involves the rapid release of NETs and appears to be independent from NADPH oxidase activity. In this pathway, an external stimulus activates a signal cascade that leads to the activation of PAD4 inducing chromatin decondensation. NE translocates into the nucleus and contributes to chromatin decondensation and nuclear membrane disintegration. In this case, however, the release of DNA and histonic proteins occurs through the formation and exocytosis of vesicles, so the cell remains alive and NETs are released externally [13] (Fig. 1).

Although NETosis and NETs have been discovered as processes responsible for capturing and killing pathogens, in agreement with the role of cancer-associated neutrophils, recent evidence suggest that these processes might play an important role in cancer progression [6, 16–18].

### NETs in cancer as diagnostic and predictive useful biomarkers

Several recent sets of evidence highlight how different circulating NET players can act as circulating biomarkers. NETs production in cancer triggers a cascade of events involving a variety of cells and blood components, including platelets, leukocytes and the tumor site itself whose outcome is to facilitate tumor progression via establishing an inflammatory microenvironment. This develops into a positive feedback loop where NETs released into the circulation cause further inflammation and activation of platelets which induce neutrophils to further release NETs. More importantly, platelet activation also promotes venous thromboembolism (VTE), one of the most dangerous effects of cancer and where recent studies suggest that NET related molecules may be useful as biomarkers of tumor-associated enhanced coagulation/thrombosis and tumor progression. In 2012, Wagner’s group showed that the release of vast amounts of DNA in the blood, putative markers of NETosis, are found at late stages of the disease and are associated with lung thrombosis in a mouse model of breast and lung syngeneic carcinoma models [19]. Recently it has been shown that the levels of circulating neutrophil proteins such as calprotectin, are useful for predicting VTE in pancreatic ductal adenocarcinoma (PDAC) and cholangiocarcinoma patients [20]. In a very elegant paper related to small intestine cancers from Rescigno’s group, the authors showed that tumor growth was mediated by increase of complement activation, enhanced NETosis and increased systemic coagulation. These events were correlated with N2 neutrophil polarization [21]. The authors concluded that a pipeline considering hypercoagulation, neutrophilia and the presence of low density neutrophils in the blood may be novel biomarkers for the early diagnosis of small intestinal tumors.

Upon initiation of NETosis, H3 becomes citrullinated by PAD4, triggering histone proteolysis that allows de-condensation of the genomic DNA to be extruded. citH3 is released into the blood from NETs and therefore represents an excellent marker of NETosis to be tested on serum. Although the clinical significance of circulating NET molecules as cancer biomarkers is still under debate, Demers’s group recently supported evidence of a direct correlation between the high levels of plasma citH3 and adverse clinical outcomes in cancer patients [22]. In this same fashion so are the studies by Decker et al., suggesting that the plasma levels of NETs, measured as granulocyte-colony stimulating factor (G-CSF) release in the blood, correlate with head and neck cancer progression [23].

Another possibility for monitoring the level of NETs released in the blood is measuring circulating cell free DNA (cfDNA) in the blood. This strategy has been found useful as a noninvasive biomarker for early diagnosis and monitoring disease progression of lung, gastroesophageal and endometrial adenocarcinomas [24–26]. NETs could be measured also in terms of a neutrophil associated protein, such as MPO and NE, bound to DNA. For example, elevated NET levels, measured as serum MPO-DNA molecules, correlates with metastatic phenotype of colorectal cancer patients [27]. Moreover, the levels of circulating NE-DNA complexes increased with the stage of the disease in breast and gastric cancers [28, 29]. Increased levels of plasma biomarkers of activated neutrophils and NETs, such as cfDNA, NE and citH3, have also recently been detected in a mouse model of pancreatic cancer [30].

In summary, so far circulating NET levels (measured as cfDNA, MPO-DNA and NE-DNA complexes as well as citH3) have been used as a surrogate of in vivo markers of neutrophil activation and NETosis in a variety of cancers. Even though in some cases their levels correlate with diagnosis and/or progression of malignancy, their role as prognostically significant biomarkers in clinical practice has still not been thoroughly demonstrated.

The main difficulty in this field arises from the fact that to date no data have been established yet in regards to the standard levels of these molecules in the blood of healthy subjects. Another aspect that should be taken into consideration, given the role played by NETosis in the presence of infections, is to have a more detailed diagnosis of the tumor of the patients enrolled in the study, excluding those who have concomitant diseases that can interfere with the cancer-associated NETosis.

Regarding resident NET molecules as cancer biomarkers, tumor-associated neutrophils as well as high levels of citH3, indicative of NETosis, have been described in the tumor microenvironment (TME). For example, in a cohort of patients undergoing liver resection for metastatic colorectal cancer, intratumoral increase NET formation, in terms of increased citrullinated histone 3, was associated with a poor prognosis [31]. Along the same line are the results provided by Yu’s group indicating that high levels of tumor-infiltrating neutrophils (TINs) and citH3 indicate poor prognosis for patients with PDAC [32]. To date tumor-associated NETs are qualitatively studied by confocal microscopy investigating specific markers such as citH3 and/or web-like extracellular DNA co-localized with MPO and NE and the development of assays allowing quantification of NETs on the tissues are still in their infancy [33, 34].

### Intratumoral NETs and NETosis in cancer progression

**Fig. 2:**
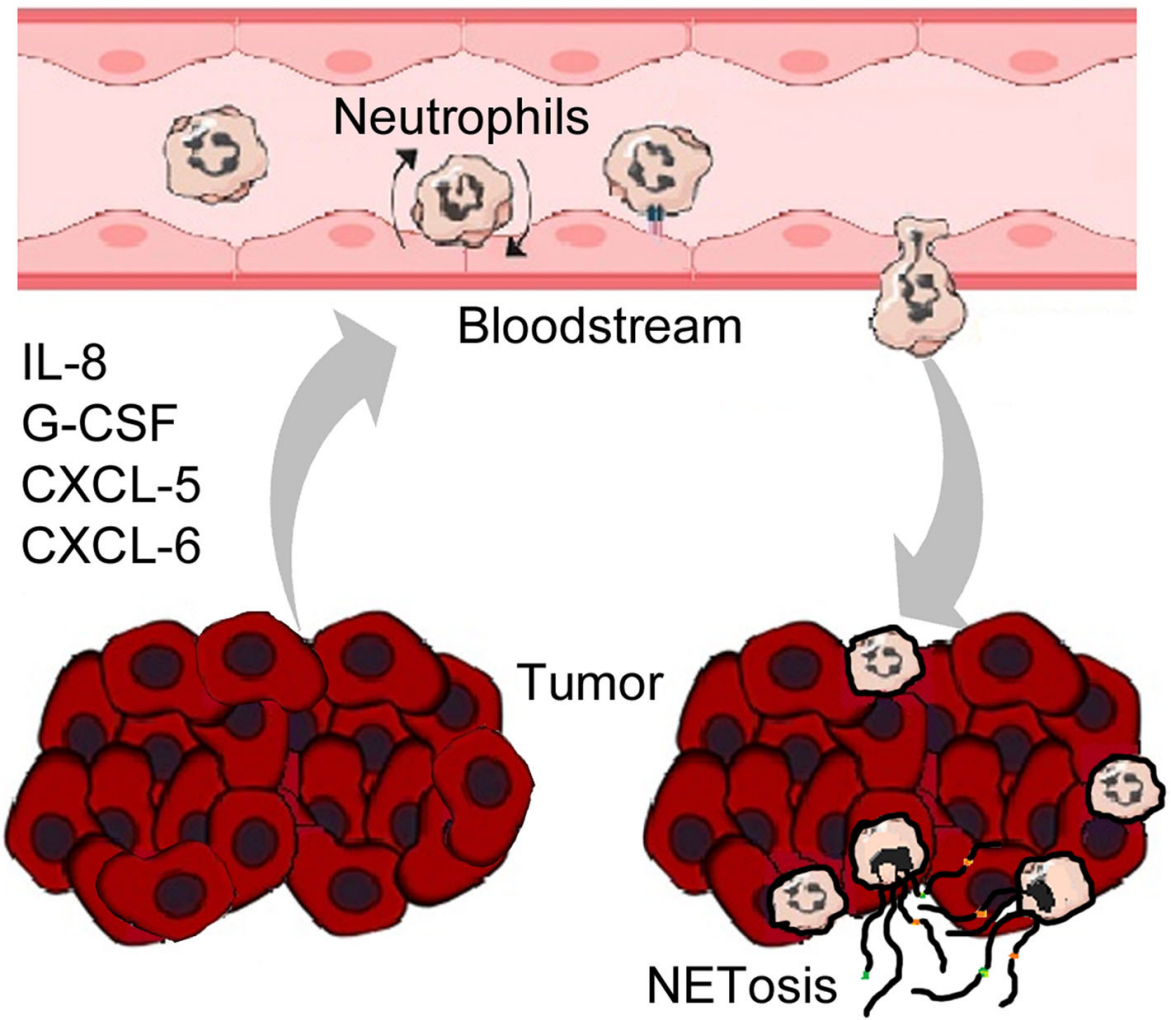
Neutrophil recall and induction of NEtosis by the tumor: Molecules released by tumors, such as chemokines (e.g. IL-8, CXCL6 and CXCL5) and G-CSF glycoprotein function as chemoattractants for neutrophils by drawing them into the tumor site from the bloodstream. Neutrophils that are recalled from the bloodstream adhere to endothelial cells, pass through gaps between endothelial cells and migrate to the tumor site. Once subjected to the influence of the tumor microenvironment, some neutrophils are induced to enter NETosis thus taking a key role in tumor progression

It is widely accepted that the immune system has a role in the development and progression of malignant tumors. In this view, a large amount of evidence attests how neutrophils make up an important component of the TME and where their controversial role in the development and progression of cancer has been widely studied over the past decade. It has been demonstrated that both neutrophilia and a higher neutrophil to lymphocyte ratio is associated with poor prognosis in several cancers [35–37] and that a crosstalk with tumor cells can cause neutrophils to switch to a pro-tumoral phenotype [38]. High neutrophil infiltration is associated with breast cancer aggressiveness and therapy resistance mediated by pro-tumor neutrophil characteristics due to the cancer cell-neutrophil interactions [39]. Tumor cells release cytokines and chemokines such as IL-8, IL-17 and G-CSF, CXCL5 and CXCL6 [18, 40] that attract neutrophils from the bone marrow to tumor sites [29, 39, 41, 42]. It has been demonstrated that significant neutrophil heterogeneity exists. Recently, Siegel’s group have shown a different role in the induction of liver metastasis from breast cancer of immature low-density and mature high-density neutrophils, the former having pro-metastatic behavior [43] (Fig. 2). Once in the TME, neutrophils are subjected to stimuli that induce NETs and NETosis and the role that the latter has in cancer progression yet what is less recognized is the role that the latter plays in cancer progression. Firstly, NETs appear to have a protective effect on cancer cells by forming a network around them. NETs appear to physically protect tumor cells from the action of cytotoxic T-lymphocytes and NK-cells by hiding the sites of interaction between effector and target cells [42]. NETs also promote the process of metastasis by contributing to migration and invasion by tumor cells [18, 24, 44]. It has been recently shown that, in gastric cancer, NETs promote a more aggressive mesenchymal phenotype [45]. Park et al. [41] demonstrated that more neutrophils were recruited in the context of murine metastatic breast cancer cells (4 T1) when compared with cells that did not metastasize. Further, the 4 T1 cell line contributed to the extensive formation of NETs, process that was successfully inhibited by DNase.

One hypothesis is that neutrophils induced to make NETosis are able to capture circulating tumor cells (CTCs), just as they are able to capture bacteria, carrying them around and thus promoting distant metastases. This mechanism has been demonstrated by Cools-Lartigue et al. [46] through a murine model in which, after inducing a condition of systemic infection that triggered NET formation, mice were injected with lung cancer cells. Regarding the molecular mechanisms underlying this process, it has recently been described the role of b1-integrin as an important factor mediating the interactions between CTCs and NETs suggesting that targeting b1-integrins may help to reduce metastases [47]. Another update hypothesis is that sustained, chronic inflammation can induce the formation of NETs, and that these play a key role in awakening dormant cancer cells. Indeed, in preclinical models where a chronic lung inflammation is induced by lipopolisaccaride or tobacco it has been demonstrated that this status is required to awaken dormant cancer cells causing metastasis in mice [48].

NETosis also appears to affect the metabolism of the tumor cell, Yazdani et al. [27] incubated isolated murine neutrophils, in which NETosis was induced, with a MC38 cell line (murine colon adenocarcinoma cells) demonstrating, in vitro, that the presence of abundant NETs in the tumor microenvironment is associated with an increase in mitochondrial function and therefore with an activation of tumor cells metabolism favoring tumor growth. Very recent studies have identified newly tumor-expressed proteins able to recruit neutrophils to the metastatic niches, inducing neutrophils to form NETs. One example is the tumor-secreted protease cathepsin C, CTSC, that have a role in promoting lung colonization of breast cancer inducing neutrophil-mediated NETs to the metastatic site [49]. Another example is the coiled-coil domain containing protein 25 (CCDC25), a protein expressed on the surface of tumor cells, that recognizes and binds the DNA molecules released by NETs. Yang et al. [50] found abundant NETs in the liver-metastases of patients with breast cancer and colon cancer and demonstrated that NET-DNA promotes human breast cancer cell migration and adhesion (MDA-MB-231 cells) through a specific interaction between CCDC25 receptor and NET-DNA [50, 51].

NETosis has also been shown to increase the severity of cancer contributing to cancer-associated thrombosis and venous thromboembolism (VTE), a major cause of mortality in cancer patients that is a fatal consequence of platelet activation [19]. The leading cause of NET involvement in coagulation is due to the fact that the DNA-protein lattice seems to constitute a scaffold for platelet adhesion and aggregation [52–54]. In addition, neutrophils and platelets adhere to each other via the glycoprotein Ibalpha expressed on the surface of platelets. This interaction activates platelets, which then expose granular P-selectin to the surface. Neutrophils recognize P-selectin via the P-selectin glycoprotein ligand-1 (PSGL-1) receptor and this platelet-neutrophil interaction facilitates NET and promote platelet aggregation and thrombus formation [55]. Indeed, it has been shown that in mice without P-selectin, platelets failed to induce NEtosis, whereas by overexpressing P-selectin in mice, platelets became even more capable of inducing NETosis [54].

### NET molecules as therapeutic targets in oncology

Based on current evidence suggesting that NETs and NETosis might play an important role in cancer progression, several working groups have recently been focusing on designing therapeutic approaches to inhibit them. Despite depleted neutrophils could be useful for cancer inhibition, this could lead to side effects due to their role in the host’s defense against pathogens. Thus, targeting NET molecules seems to be a more suitable approach in cancer treatment. One candidate to be used to inhibit NETosis is the DNAse I, a treatment already approved by the Food and Drug Administration (FDA) for the treatment of cystic fibrosis leading to a decrease of NET accumulation in the patient lungs [56]. Besides DNAse I treatment, small molecules inhibiting NET components have been used in preclinical models. Pharmacological inhibition of PAD4, the enzyme that mediates NETs formation from neutrophils, has been used in several animal models of cancer. In mice, the use of this drug prevents renal dysfunction associated with breast and pancreatic cancer. Moreover, the authors identify NETosis as a cause of cancer-associated renal failure [57]. Similarly, in a mouse model of lung metastasis from breast cancer anti-NET therapies such as DNase I or PAD inhibitors interfere with the process of metastasis [58]. NET-targeted therapies based on inhibition of NET formation by genetic alteration (PAD4 knockout mice), pharmacology inhibition of NE, or DNase1 treatment in preclinical models leads to decreased lung and colon cancer invasion [24] (Table 1).

**Table 1:** CXCR1/2, PAD4 inhibitors and DNase in preclinical models

PRECLINICAL MODELS OF CANCER			
Target	Inhibitor/enzyme	Tumor	References
CXCR1/2	Reparixin	Breast cancer	[42]
PAD4/PD-1/CTLA-4	GSK484/ anti-PD-1 and anti-CTLA-4 checkpoint inhibitors	Breast cancer	[42]
PAD4/ DNA	GSK484/ DNase	Mammary carcinoma (MMTV-PyMT) and pancreatic tumors (RIP1-Tag2)	[57]
PAD4/ DNA	Cl-amidine/ DNase	Lung metastasis	[58]
PAD4/DNA/ NE	PAD4^−/−^ /DNase / Sivelestad	Lung and colon cancer invasion	[24]
PAD4/PD-1	PAD4^−/−^ /anti-PD-1 checkpoint inhibitor	Pancreatic tumor (PDAC)	[59]

(Reparixin) CXCR1/2 inhibitor; (GSK484) PAD4 inhibitor; (CI-amidine) PAD inhibitor; (Silvelestad) NE inhibitor; (MMTV-PyMT) mouse model of breast cancer metastasis; (PDAC) pancreatic ductal adenocarcinoma; (RIP1-Tag2) mouse model of β-cell carcinogenesis

In addition to inhibiting NETs and NETosis per se, many efforts have recently been made to understand whether the combination of their inhibition with current therapies can be successful. Interestingly, some studies have been done in the area of immunotherapy, combining NETs with immune checkpoint inhibitors. Immune checkpoint blockade reverses immune suppression to activate tumor-reactive cytotoxic T lymphocytes (CTLs) in the TME that directly target tumor cells for apoptosis. However, despite exciting progress, the benefits of these therapies in patients with advanced malignancy have been limited. Thus, there is a growing interest in understanding whether neutrophil inhibition strategies are able to overcome immunotherapy resistance [60]. The rationale resides in the fact that the TME contributes in promoting tumor progression and includes, among others, neutrophils that represent a high proportion of the immune infiltrate in several cancer types [38]. Interestingly, a high number of tumor-associated neutrophils in the TME and aberrant NETs release were reported to be associated with poor response to immunotherapy in several cancers thus making neutrophils and NETs molecules interesting targets for and a mechanism of resistance to these drugs [60]. The idea is that if we simultaneously inhibit the aberrant innate immune response, fueled by tumor cells, and the checkpoints of the acquired immune response, fueled by both the innate response and tumor cells, we may be able to overcome the drug resistance observed with inhibition of the immune checkpoint alone. In a very elegant paper, McAllister‘s group has shown that inhibiting the neutrophil tumor recruitment, through their in vivo depletion, in an orthotopic mouse model of PDAC make tumor cells more sensitive to immunocheckpoint drug inhibition. Of note, they have also demonstrated a direct involvement of NETosis in this process, using a recipient animal model in which the PAD4 gene is deleted. In this context, NET is inhibited and PDAC responds much better to the immunocheckpoint inhibitors than in mice expressing PAD4 and making NETs in the TME [59]. In the same manner, are recently reported results combining anti-PD-1 and anti-CTLA-4 checkpoint inhibitors with pharmacological inhibition of PAD4 in a syngeneic mouse model of breast cancer and metastasis based on subcutaneous and intravenously injected 4 T1 cells [42]. In this experimental setting, the authors demonstrated that tumor-secreted CXCR1 and CXCR2 ligands, Interleukin 8 (IL-8 or CXCL-8), induce extrusion of NETs protecting tumor cells from CTL and the natural killer cell (NK) cytotoxicity. Of note, this result indicates that NETs impair contact of immune cytotoxic cells with tumor cell inhibition of NETosis sensitizing tumors to checkpoint inhibitors.

In addition to the above preclinical studies, some clinical trials are currently ongoing and are testing the efficacy of inhibiting NETs simultaneously with other therapeutic strategies. Based on the importance of the neutrophil/NET axis, CXCR1/2 and the IL-8 pathway have attracted a lot of interest as therapeutic targets. Several CXCR1 and 2 inhibitors have already been tested in clinical trials in combination with immune checkpoint inhibitors. A phase I study in advanced melanoma patients is evaluating the CXCR1/2 inhibitor, SX-682, in combination with the monoclonal antibody anti-PD1, pembrolizumab (NCT03161431). A phase II study in advanced solid tumors is evaluating the CXCR1/2 inhibitor Navarixin in combination with pembrolizumab, as well (NCT03473925)(Table 2).

**Table 2:** Active clinical trials in tumors with CXCR1 and CXCR2 inhibitors

	ACTIVE CLINICAL TRIALS IN TUMORS			
Target	inhibitor	Pathology	N° of trial	Phase
CXCR2	AZD5069 in combination with the androgen receptor antagonist, Enzalutamide	Metastatic Castration Resistant Prostate Cancer	NCT03177187	2
CXCR1/2	SX-682 in combination with the anti-PD-1, Nivolumab	RAS Mutated Microsatellite Stable Metastatic Colorectal Cancer	NCT04599140	2
CXCR1/2	SX-682 in combination with the anti-PD-1, Nivolumab	Metastatic Pancreatic Ductal Adenocarcinoma	NCT04477343	1
CXCR1/2	SX-682 in combination with the bifunctional fusion protein targeting TGF-β and PD-L1 (BinTrafusp Alfa or M7824) and with the cancer vaccine CV301 TRICOM targeting carcinoembryonic antigen (CEA) and mucin1 protein (MUC1)	Advanced Solid Tumors (STAT)	NCT04574583	2
CXCR1/2	SX-682 in combination with the anti-PD-1, Pembrolizumab	Metastatic Melanoma	NCT03161431	1
CXCR1/2	Navarixin in combination with the anti-PD-1, Pembrolizumab	Advanced/Metastatic Solid Tumors (Non-small Cell Lung Cancer, Castration Resistant Prostate Cancer, Microsatellite Stable Colorectal Cancer)	NCT03473925	2

(AZD5069) CXCR2 antagonis; (Navarixin) CXCR1/2 inhibitor; (Pembrolizumab) monoclonal antibody anti-PD1; (SX-682) CXCR1/2 inhibitor

## Conclusions

In conclusion, based on the data available to date, it is time we start to believethat future analyses of the processes related to NETosis should become standard diagnostic and prognostic routine in the cancer clinic. It is also time to imagine a scenario in which different NETosis stages/molecules may be key targets of the activity of drugs for the treatment of cancer.

## Data Availability

“Not applicable”.

## Abbreviations

AZD5069: CXCR2 antagonist
CCDC25: coiled-coil domain containing protein 25
citH3: citrullinated histone H3
CR: complement receptor
CTCs: circulating tumor cells
CTLs: cytotoxic T lymphocytes
CTSC: tumor-secreted protease cathepsin C
CXCR1: Interleukin 8 receptor alpha or C-X-C motif chemokine receptor 1
CXCR2: Interleukin 8 receptor beta or C-X-C motif chemokine receptor 2
FDA: Food and Drug Administration
G-CSF: granulocyte-colony stimulating factor
IL-8 or CXCL-8: Interleukin 8
LPS: bacterial lipopolysaccharides
MMTV-PyMT: mouse model of breast cancer metastasis
MPO: myeloperoxidase
Navarixin: CXCR1/2 inhibitor
NE: serine protease neutrophil elastase
NET: neutrophil extracellular trap
NK: natural killer cell
PAD4: peptidyl arginine deiminase 4
PDAC: pancreatic ductal adenocarcinoma
Pembrolizumab: monoclonal antibody anti-PD1
PMA: phorbol 12-myristate 13-acetate
PSGL-1: P-selectin glycoprotein ligand-1
RIP1-Tag2: mouse model of β-cell carcinogenesis
ROS: reactive oxygen species
SX-682: CXCR1/2 inhibitor
TILs: tumor infiltrating lymphocytes
TINs: tumor-infiltrating neutrophils
TLR: toll–like receptor
TMB: tumor mutation burden
TME: tumor microenvironment
VTE: venous thromboembolism
